# Ticagrelor-Induced Complete Heart Block

**DOI:** 10.7759/cureus.95430

**Published:** 2025-10-26

**Authors:** Zubair Farooq, Saurabh kumar Singh, Shrividya Rao, Anwar Ansari, Devesh Kumar

**Affiliations:** 1 Cardiology, Vardhman Mahavir Medical College and Safdarjung Hospital, Delhi, IND; 2 Cardiology, Vardhman Mahavir Medical College and Safdarjung Hospital, New Delhi, IND; 3 Medicine, Atal Bihari Vajpayee Institute of Medical Sciences and Dr. Ram Manohar Lohia Hospital, New Delhi, IND; 4 Cardiothoracic Surgery, All India Institute of Medical Sciences, New Delhi, New Delhi, IND

**Keywords:** complete heart block, pacemaker, reversible, ticagrelor, transient

## Abstract

A young male in his mid-30s presented with acute onset of severe, retrosternal chest pain of 12 hours’ duration. 12-lead electrocardiogram showed ST-segment elevation in chest leads V1-V6, confirming a diagnosis of anterior wall myocardial infarction (AWMI). He was taken up for urgent coronary angiography, which revealed a proximal left anterior descending (LAD) thrombotic lesion (other coronaries being completely normal). Primary percutaneous coronary intervention (PCI) was performed, deploying a 3.5×33 mm stent from the LAD artery ostium, with TIMI 3 flow. The patient was initiated on dual antiplatelet therapy with aspirin and ticagrelor. On day two, the patient experienced multiple episodes of syncope and was found to have complete heart block (CHB) with a heart rate dropping to 30 beats per minute. A temporary transvenous pacemaker was inserted, and after ruling out all possible reversible etiologies, a check angiogram was done. It revealed a patent stent with TIMI 3 flow in the LAD, and all other vessels were normal. Ticagrelor was suspected as a plausible cause of CHB and was substituted with clopidogrel. This was followed by an intermittent return to sinus rhythm within 24 hours and complete resolution of CHB by 40 hours with no further episodes of CHB occurring after 48hours of discontinuation of ticagrelor. This case highlights the potential for ticagrelor-induced CHB even in young individuals without any preexisting conduction system disease and the importance of early recognition and prompt management, which can prove to be lifesaving.

## Introduction

Dual antiplatelet therapy is imperative in the management of patients following acute coronary syndromes and percutaneous coronary intervention (PCI) [1]. Ticagrelor, a reversible P2Y₁₂ receptor antagonist, has demonstrated superior efficacy over clopidogrel in reducing thrombotic events in patients with acute coronary syndromes (ACS). The Platelet Inhibition and Patient Outcomes (PLATO) trial depicted that ticagrelor, in conjunction with low-dose aspirin, significantly decreased the rates of death from vascular causes, myocardial infarction, or stroke compared to clopidogrel [2]. The superior efficacy of ticagrelor over other P2Y12 antagonists is partly attributed to its pleiotropic effects, which increase adenosine levels and contribute to cardio-protection, anticoagulation, and anti-inflammatory properties. However, these properties may also lead to significant adverse effects, including electrophysiological disturbances [3]. Although bradycardia associated with ticagrelor is typically transient and asymptomatic, severe manifestations such as complete heart block (CHB) are rare and under-reported.

No part of this article has been presented at any conference or published in any conference prior to this submission.

## Case presentation

**Figure 1:**
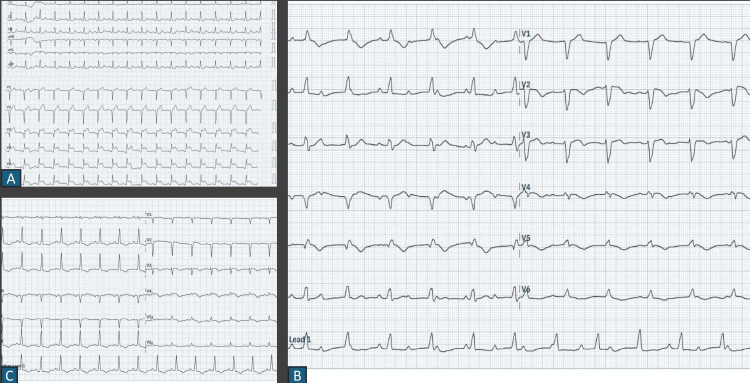
Part A shows a 12-lead electrocardiogram with ST-segment elevation in V1-V6 consistent with a diagnosis of anterior wall myocardial infarction. Part B shows a 12-lead electrocardiogram with complete heart block and A-V dissociation. Part C shows resolution of the complete heart block with return to sinus rhythm and q waves in the anterior leads.

A young male in his mid-30s, with no known medical comorbidities, presented to the emergency department with a 12-hour history of retrosternal chest pain, radiating to the left arm. He described the pain as severe, constant, associated with diaphoresis and shortness of breath. The patient had no history of similar symptoms, prior heart disease, or family history of events. On arrival, vital signs were notable for a heart rate of 110 beats per minute, blood pressure of 90/60 mmHg, and respiratory rate of 24 breaths per minute. The patient also had bilateral basilar, end-inspiratory crepts with raised Jugular Venous Pressure (Killip’s Class II). 12-lead electrocardiogram (ECG) revealed significant ST-segment elevation in leads V2 through V6, suggesting an anterior wall myocardial infarction (Figure 1A).

**Figure 2:**
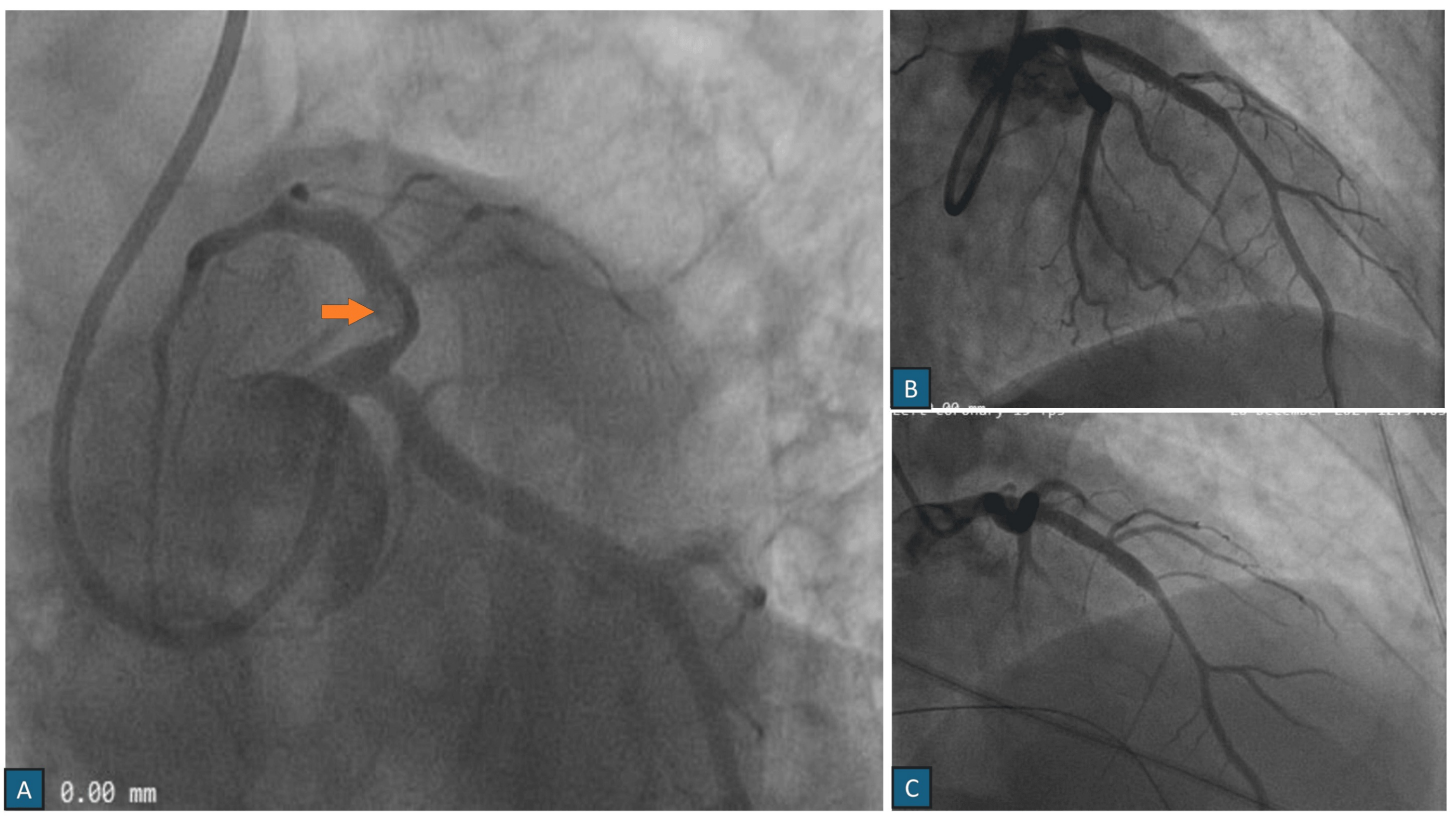
Part A shows a coronary angiogram of the left system in the Left Anterior Oblique caudal view showing a Grade III thrombus in the proximal Left Anterior Descending (LAD) artery with an eccentric plaque leading to 80% stenosis in proximal LAD (Marked with Orange Arrow). Part B shows LAD in the Right Anterior Oblique Cranial view following stenting, and Part C shows the check angiography in the Right Anterior Oblique Cranial View with TIMI 3 flow.

Subsequently, the patient was taken for urgent primary PCI. The proximal left anterior descending artery (LAD) was found to be obstructed along with Grade III thrombus, and a 3.5 × 33 mm drug-eluting stent was successfully deployed. The procedure resulted in TIMI 3 flow with no other significant coronary pathology (Figures 2A-2B).

Following PCI, the patient was administered dual antiplatelet therapy with Ecosprin 75 mg and ticagrelor 90 mg BD after loading doses of Ecosprin 300mg and ticagrelor 180 mg, respectively. Other medications included atorvastatin 80 mg and ramipril 2.5 mg. On day two of his hospital stay, the patient suddenly developed dizziness and was noted to have a heart rate of 30 beats per minute on continuous monitoring, with associated CHB (Figure 1B). 12-lead electrocardiogram confirmed the diagnosis of CHB, and subsequently, a transvenous temporary pacemaker was inserted to stabilize the patient. The work-up, including assessment of electrolytes, creatinine clearance, liver enzymes, and thyroid function, was all within normal limits. A check angiogram showed a patent stent with TIMI 3 flow in the LAD, ruling out a coronary etiology for the conduction disturbance (Figure 2C). The patient reported taking ticagrelor about an hour prior to the onset of symptoms, which raised suspicion for ticagrelor-induced CHB. Given the timing of onset of symptoms and the exclusion of other potential correctable causes, ticagrelor was substituted with clopidogrel.

The transvenous temporary pacemaker was kept in situ for 72 hours, with resolution of CHB to normal sinus rhythm intermittently seen at 24 hours and complete resolution to sinus rhythm within 48 hours after cessation of ticagrelor (Figure 1C). Once the patient was in normal sinus rhythm for 48 hours with no conduction defects, the temporary pacemaker was removed. The patient was discharged subsequently on guideline-directed medical therapy with a low dose of oral beta-blocker started 48 hours prior to discharge.

The patient was monitored closely in the coronary care unit (CCU) during his hospital stay, with continuous rhythm monitoring using a cardiac monitor. After the temporary pacemaker was removed, there were no further episodes of heart block, bradycardia, or sinus pauses. The patient was discharged on day seven post-procedure, with instructions to continue dual antiplatelet therapy for a total duration of 12 months [4].

At discharge, the patient was advised to avoid strenuous physical activity for at least 2 weeks and to follow up in the cardiology outpatient clinic within 1 week for further evaluation. During his follow-up visit, the patient was clinically stable, with a normal heart rate and rhythm, and was resuming normal activities without any recurrence of symptoms. ECG showed a normal sinus rhythm without signs of conduction disturbances, and a 24-hour Holter recording failed to show any transient sinus pauses or conduction system disease.

## Discussion

Ticagrelor is a potent oral P2Y12 receptor antagonist that binds reversibly to prevent ADP-mediated platelet activation and aggregation. Unlike clopidogrel, ticagrelor does not require metabolic activation and has a faster onset, greater potency, and reduced response variability [5]. In addition to its potent antiplatelet effects, ticagrelor is associated with distinctive adverse effects, including dyspnea and ventricular pauses, which are thought to result from its ability to delay adenosine metabolism by inhibiting the equilibrative nucleoside transporter (ENT)-1. These side effects, often manifesting as early-onset dyspnea and sinoatrial node pauses, are typically self-limiting and rarely necessitate discontinuation of the drug [6]. Bradycardia associated with ticagrelor was initially identified in a phase IIb dose-ranging study, where post hoc analysis revealed an unexpected increase in predominantly asymptomatic ventricular pauses [3,5]. This observation was further supported by the prospective PLATO trial [4,6]. Among 2908 patients who underwent 7-day electrocardiographic monitoring at randomization and again at 1 month, ventricular pauses lasting ≥3 seconds were more common in those receiving ticagrelor compared to clopidogrel during the first week (5.8% vs 3.6%, relative risk 1.61, P =.006). These pauses were mostly asymptomatic, of sinoatrial origin, and occurred nocturnally. Importantly, no significant difference in brady-arrhythmias was noted at 1 month, and no clinically significant bradycardic events were reported during follow-up [7].

In our case, the patient was a young 37-year-old who developed CHB without any evidence of conduction system disease on baseline electrocardiogram (ECG). This presentation is particularly unusual, as most documented cases of ticagrelor-induced atrioventricular (AV) block have occurred in older patients, who are more likely to have preexisting conduction abnormalities or structural heart disease. For instance, three previously reported cases of ticagrelor-associated AV block involved patients aged 75, 81, and 61 years, respectively, suggesting an age-related predisposition or an underlying vulnerability in the conduction system [7-9]. The reported cases in the literature with CHB have been compiled below (Table 1).

**Table 1:** Summary of case reports on ticagrelor-induced complete heart block AWMI: anterior wall myocardial infarction; NSTEMI: non-ST-elevation myocardial infarction

Author (Year)	Age/Sex	Presenting Condition	Baseline ECG	Time to CHB After Ticagrelor	Management	Reversibility
Goldberg et al. 2015 [11]	52 years/M	NSTEMI	Right bundle branch block	Four hours	Ticagrelor stopped and temporary pacemaker inserted	Yes
Yurtdas and Ozdemir 2017 [12]	47 years/F	NSTEMI	No baseline conduction abnormality	10 hours	Ticagrelor stopped and substituted with Prasugrel	Yes
Waldman et al. 2018 [7]	75 years/F	NSTEMI	No baseline conduction abnormality	Few hours	Ticagrelor stopped and substituted with clopidogrel	Yes
Premchand et al. 2020 [8]	81 years/M	Chronic stable angina	Right bundle branch block	5 days	Ticagrelor stopped and substituted with clopidogrel	Yes
Anand et al. 2020 [13]	70 years/M	Unstable angina	Left bundle branch block	1 week	Ticagrelor stopped and substituted with clopidogrel	Yes
Trivedi et al. 2021 [14]	50 years/M	AWMI	No conduction abnormality	Day 2	Ticagrelor stopped and substituted with Prasugrel	Yes

In a study involving seven ACS patients with severe bradyarrhythmia, six of the patients had preexisting conduction system abnormalities, including first-degree AV block, left bundle branch block (LBBB), or right bundle branch block (RBBB), which are known risk factors for developing high-degree AV block. Three patients required temporary pacing due to severe symptoms, and two patients with persistent high-degree AV block (one with a long PR interval and the other with baseline RBBB and left anterior hemiblock) had permanent pacemakers implanted. Additionally, five of the seven patients were taking beta-blocker therapy, a factor that increases the risk of brady-arrhythmias. Notably, four of these patients had diabetes, which has been associated with a higher incidence of cardiac conduction abnormalities, even sub-clinically. In the PLATO trial, 25% of the population had diabetes, although preexisting conduction system disease was not clearly reported. The PLATO sub-study investigating brady-arrhythmias found that most patients with ventricular pauses were also on beta-blocker therapy [10].

The absence of any conduction system disease in our patient highlights the potential for ticagrelor to induce significant electrophysiological disturbances even in individuals without apparent risk factors, raising questions about the underlying mechanisms in such atypical presentations.

In light of the potential for ticagrelor to cause significant conduction disturbances in younger, otherwise healthy individuals, clinicians should maintain a high index of suspicion when monitoring patients, especially in the early stages of therapy. While adverse events such as bradyarrhythmia are relatively rare and often self-limiting, early detection and careful management, including electrocardiographic monitoring, may help mitigate the risk of severe complications such as CHB or the need for permanent pacing.

Given the limited data on ticagrelor-induced conduction disturbances in young patients, further studies are warranted to better define the clinical and electrophysiological characteristics of such cases. A deeper understanding of risk factors, genetic predisposition, or pharmacodynamic interactions may help predict which patients are at higher risk and guide more personalized therapeutic approaches.

## Conclusions

Ticagrelor may cause paroxysmal CHB, a rare but potentially life-threatening complication, even in young patients without preexisting conduction system disease, particularly in the early stages of therapy after PCI. Early detection and monitoring of patients on ticagrelor, especially in the ICU post-PCI, is crucial for identifying arrhythmic complications, including bradycardia and AV block. Temporary pacing should be considered for symptomatic bradycardia or CHB until the drug is adequately cleared from the system (4-5 half-lives), with close monitoring for recurrence. Switching from ticagrelor to clopidogrel or prasugrel in patients with suspected drug-induced CHB is an effective management strategy, as they do not have the same adenosine-like effects on the conduction system.
